# Using *Vibrio natriegens* for High-Yield Production of Challenging Expression Targets and for Protein Perdeuteration

**DOI:** 10.1021/acs.biochem.3c00612

**Published:** 2024-02-15

**Authors:** Natalia Mojica, Flore Kersten, Mateu Montserrat-Canals, G. Robb Huhn III, Abelone M. Tislevoll, Gabriele Cordara, Ken Teter, Ute Krengel

**Affiliations:** † Department of Chemistry, University of Oslo, NO-0315 Blindern, Oslo, Norway; ‡ Centre for Molecular Medicine Norway, University of Oslo, NO-0318 Blindern, Oslo, Norway; § Burnett School of Biomedical Sciences, College of Medicine, University of Central Florida, Orlando, Florida 32816, United States

## Abstract

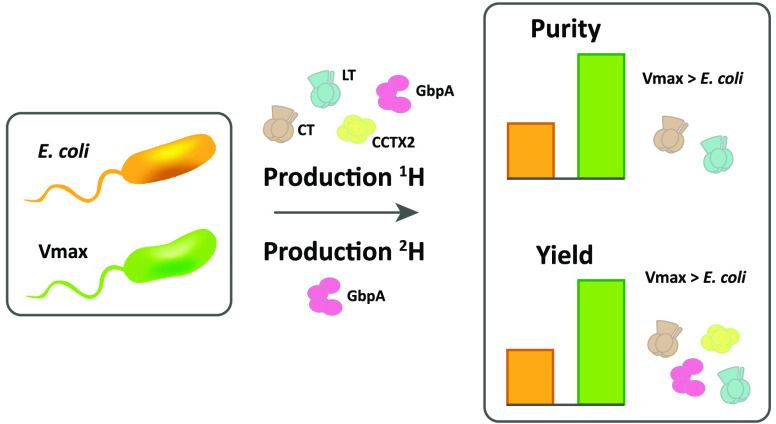

Production of soluble proteins is essential for structure/function studies; however, this usually requires milligram amounts of protein, which can be difficult to obtain with traditional expression systems. Recently, the Gram-negative bacterium *Vibrio natriegens* emerged as a novel and alternative host platform for production of proteins in high yields. Here, we used a commercial strain derived from *V. natriegens* (Vmax X2) to produce soluble bacterial and fungal proteins in milligram scale, which we struggled to achieve in *Escherichia coli*. These proteins include the cholera toxin (CT) and *N*-acetyl glucosamine-binding protein A (GbpA) from *Vibrio cholerae*, the heat-labile enterotoxin (LT) from *E. coli* and the fungal nematotoxin CCTX2 from *Coprinopsis cinerea*. CT, GbpA, and LT are secreted by the Type II secretion system in their natural hosts. When these three proteins were produced in Vmax, they were also secreted and could be recovered from the growth media. This simplified the downstream purification procedure and resulted in considerably higher protein yields compared to production in *E. coli* (6- to 26-fold increase). We also tested Vmax for protein perdeuteration using deuterated minimal media with deuterium oxide as solvent and achieved a 3-fold increase in yield compared to the equivalent protocol in *E. coli*. This is good news, since isotopic labeling is expensive and often ineffective but represents a necessary prerequisite for some structural biology techniques. Thus, Vmax represents a promising host for production of challenging expression targets and for protein perdeuteration in amounts suitable for structural biology studies.

Obtaining structural information on toxins and other virulence factors from bacteria and fungi is essential to understand the molecular mechanisms behind infections. Structural biology studies often rely on the production of the target protein in high amounts (milligram scale), which is usually achieved by expression in *Escherichia coli*.^1^ This Gram-negative bacterium is the first choice for protein production in many laboratories due to its fast growth in inexpensive media, its well-characterized genome, and the availability of a wide range of commercial *E. coli* strains for different applications.^2^ However, expression yields are not always sufficient; in addition, other challenges abound, such as formation of insoluble aggregates.^3^*E. coli* strains can also be used for the production of deuterated and perdeuterated proteins, which are highly useful for neutron scattering techniques, such as neutron crystallography and small-angle neutron scattering (SANS)^4^ but also NMR spectroscopy.^5^ In deuterated proteins, a significant number of the hydrogen atoms are substituted for deuterium; perdeuteration refers to the almost complete substitution of hydrogen atoms for deuterium. Deuterated reagents are expensive, highlighting the importance of an expression system that can efficiently produce high yields of protein. Overcoming challenges related to protein production often requires significant optimization of the growth conditions, modification of the constructs, or the use of different bacterial strains, which is a time-consuming process and not always successful.

As an alternative to *E. coli*, a new potential expression host has emerged: *Vibrio natriegens*.^6^ This Gram-negative marine bacterium can grow twice as fast as *E. coli* under optimal conditions.^7^ Such rapid growth requires a very efficient protein synthesis machinery, which has been mainly attributed to the higher number of ribosomes produced by *V. natriegens* in the exponential phase: 115 000 versus 70 000 in *E. coli*.^8^ In addition, *V. natriegens* has its genome distributed between two chromosomes; thus, replication can occur in parallel from two origins of replication.^9^ Its fast growth and ability to rapidly synthesize proteins makes *V. natriegens* an interesting species for protein production. In 2017, an engineered strain known as Vmax X2 (originally Vmax Express; here referred to as Vmax) was made commercially available for the expression of genes under the control of an arabinose or isopropyl β-d-1-thiogalactopyranoside (IPTG)-inducible T7 promoter, allowing the use of *E. coli*-based plasmids in *V. natriegens*.^6^ Since then, different proteins have been produced using Vmax, including membrane proteins from *Vibrio cholerae*,^10^ recombinant enzymes cloned into pET vectors,^11^ and an insect metalloproteinase inhibitor fused to *N*-acetylglucosamine-binding protein A (GbpA),^12^ which was used as a secretion tag. In addition, Vmax has been used for ^15^N-labeling.^13^ To the best of our knowledge, *V. natriegens* has not yet been used for protein deuteration.

In our own work, we have experienced on a number of occasions the limitations of *E. coli* for producing soluble, active proteins in sufficient amounts for structural studies. One problematic target is the cholera toxin (CT), the main virulence factor of *V. cholerae*. CT is composed of a catalytic A-subunit (CTA) and a homopentamer of cell-binding B-subunits (CTB) that are assembled to an AB_5_ toxin in the periplasm. Thereafter, the multimeric toxin is secreted into the extracellular environment by the *V. cholerae* type II secretion system (T2SS).^14−16^ When produced in *E. coli*, we could not recover CT from the growth medium. Only low yields of toxin (0.2 mg per L culture) were obtained from the periplasmic space, in a mixture of intact holotoxin and free CTB. We reasoned that recombinant CT would be secreted by the *V. natriegens* T2SS,^12,16^ which could improve holotoxin yield and purity. *V. natriegens* therefore appeared like a suitable system to produce CT. In addition, we tested production of two other proteins secreted by the T2SS: GbpA from *V. cholerae* and heat-labile enterotoxin (LT) from enterotoxigenic *E. coli* (ETEC). We also tested *V. natriegens*—with success—for protein perdeuteration, which produced increased yields when compared to *E. coli* expression systems, opening the door to neutron-based techniques.^4^ In a final test, we extended the work to nonsecreted proteins, producing a fungal nematotoxin from *Coprinopsis cinerea* (CCTX2).^17^ When compared to expression in *E. coli*, Vmax significantly improved the yields of all tested proteins, and in some cases considerably simplified the purification process. *V. natriegens* thus represents a promising system for the production of challenging protein targets.

## Materials and Methods

### Constructs and Bacterial Strains

Plasmids encoding CT, LT, GbpA, and *C. cinerea* Toxin 2 (CCTX2) were used for protein production in both *E. coli* and Vmax. N-terminal signal sequences directed CT, LT, and GbpA to the periplasmic space, where they were cleaved from the mature proteins, whereas CCTX2 remains in the cytoplasm of the bacterial expression hosts.

Professor Randall K. Holmes kindly provided us with a pARCT5 vector (derived from the pAR3 vector)^18^ encoding the *ctxAB* operon under the control of an l-arabinose-inducible promoter (Figure S1A). The translated genes contained the signal sequence from *E. coli* LT-IIB. This tag directs CTA and CTB to the periplasmic space more efficiently than their native CT secretion signals.^19^

The operon encoding LT from ETEC strains of porcine origin (also known as pLT) was synthesized by Genscript (Leiden, Netherlands) with LT-IIB signal sequences, provided in a pUC57 vector, and subcloned into a mutated version of pARCT5 containing only two (instead of three) NcoI restriction sites. These restriction sites were used to replace the DNA coding for CTA and CTB with the coding sequences for LTA and LTB (i.e., *eltA* and *eltB*), respectively (Figure S1B).

The gene encoding GbpA (Uniprot ID: Q9KLD5, residues 24–485) was codon-optimized for *E. coli* and cloned into pET-26b(+) by GenScript (Leiden, Netherlands) using restriction sites NcoI and XhoI. The natural signal peptide of the protein was substituted by the pelB leader sequence present in the vector, which in *E. coli* signals the protein for periplasmic localization but in Vmax directs the secretion of the protein into the culture media (Figure S1C). The vector originally contained a C-terminal His_6_-tag, but introduction of a stop codon at the end of the insert prevented expression of the tag.

For CCTX2, vector pET-24b(+), which contains the wild-type *cctx2*-gene under control of an IPTG-inducible T7 promoter (Figure S1D), was provided by Professor Markus Künzler (ETH Zurich).

A summary of the constructs and strains used in this work is shown in Table 1.

**Table 1:** Summary of Constructs and Strains Used for Protein Production

Gene	Protein	Construct^a^	Antibiotic resistance gene	Strains	Protein’s subcellular location	Growth media^b^
*ctxAB*	CT	pARCT5	Chloramphenicol	*E. coli:* Overexpress C43 (DE3)	Periplasm	TB
				*V. natriegens*: Vmax	Secreted	LB-v2 salts
*eltAB*	LT	pARpLT5	Chloramphenicol	*V. natriegens*: Vmax	Secreted	LB-v2 salts
*gbpA*	GbpA	pET-26b(+)	Kanamycin	*E. coli*: BL21 Star (DE3)	Periplasm	M9glyc+
				*V. natriegens*: Vmax	Secreted	M9max
*cctx2*	CCTX2	pET-24b(+)	Kanamycin	*E. coli*: Overexpress C41 (DE3)	Cytosol	LB
				*V. natriegens*: Vmax	Cytosol	LB-v2 salts

a Plasmid maps are shown in Figure S1.

b A detailed description of the M9glyc+ and M9max media is found in the Supporting Information (Tables S1 and S2).

### Transformation

All plasmids were introduced into chemically competent Vmax cells (TelesisBio, San Diego) by heat shock, following the manufacturer’s guidelines. Briefly, 100–200 ng of plasmid DNA was incubated with Vmax competent cells on ice for 30 min before heat shock at 42 °C for 45 s in a water bath. The cells were immediately returned to ice for 2–5 min and then transferred to a prewarmed 14 mL Falcon tube containing 950 μL of Vmax chemicompetent cell recovery medium and incubated at 30 °C for 2 h in a shaker. Cells were plated on Luria–Bertani (LB) agar plates containing the appropriate antibiotics (12.5 μg/mL chloramphenicol (CAM) for cells transformed vectors encoding CT and LT, or 100 μg/mL kanamycin (Kan) for GbpA and CCTX2-encoding vectors) and incubated overnight at 30 °C. Single colonies were picked to grow overnight cultures in LB+v2 medium (LB media supplemented with 204 mM NaCl, 4.2 mM KCl, and 23.1 mM MgCl_2_ (v2 salts))^20^ with corresponding antibiotics. Subsequently, we prepared glycerol stocks that were stored at −80 °C.

### Recombinant Expression in *E. coli*

#### Expression of ctxAB

Expression was essentially performed as described previously.^21^ Briefly, OverExpress C43 (DE3) cells (Sigma) harboring pARCT5 were grown overnight at 30 °C in Terrific Broth (TB) medium containing 25 μg/mL CAM. Cultures were diluted 1/50 in TB medium, grown until the optical density at 600 nm (OD_600_) reached 2.0, and induced with 0.2% l-arabinose (Sigma) at 37 °C. After 3 h, the cells were harvested by centrifugation (6000*g*, 20 min, 4 °C) and resuspended in 1/40th volume of Talon A buffer (50 mM sodium phosphate pH 8.0, 300 mM NaCl), supplemented with c*O*mplete^TM^ protease inhibitor cocktail (Roche), 1 mg/mL polymyxin B sulfate (Sigma), and benzonase (Sigma). This solution was incubated at 37 °C for 15 min with shaking followed by centrifugation (8000*g*, 20 min, 4 °C). The supernatant (containing the periplasmic extract) was filtered through a 0.22 μm filter (polyethersulfone (PES) membrane, VWR) and used immediately for further purification.

#### Expression of gbpA

GbpA production was carried out essentially as described by Sørensen et al.^22^ BL21 Star (DE3) cells transformed with the codon-optimized GbpA-encoding pET-26b(+) vector were grown for 6 h at 37 °C, 220 rpm, in 2.5 mL LB medium containing 50 μg/mL Kan (until OD_600_ ≈ 4). 200 μL of the preculture was diluted in 25 mL (1/125 dilution) minimal medium M9glyc+^23^ (see Table S1 for recipe) and grown for 14 h at 37 °C, 130 rpm (to OD_600_ ≈ 13). The main culture was started by adding 225 mL of M9glyc+ medium to the culture and adjusting the concentration of antibiotic back to 50 μg/mL Kan. When OD_600_ reached 2 to 3, IPTG was added to a final concentration of 1 mM to induce expression. After incubation for 18 h at 20 °C, 130 rpm, cells were harvested by centrifugation at 10 000*g* for 30 min at 4 °C. GbpA was isolated from the periplasmic space by osmotic shock. Briefly, the pellet was resuspended in 5 mL sucrose solution (25% w/v sucrose, 20 mM Tris-HCl pH 8.0, 5 mM ethylenediaminetetraacetic acid (EDTA)) per gram of cells. The cell suspension was incubated on ice for 30 min under stirring, followed by centrifugation at 10 000*g* for 30 min at 4 °C. The supernatant was saved as sucrose fraction for further processing. The pellet was resuspended in 5 mL per gram of cells of a hypotonic solution (5 mM MgCl_2_, 0.25 mg/mL lysozyme from chicken egg white >40 000 units/mg protein (Sigma), and 1 mM phenylmethylsulfonyl fluoride). The suspension was again incubated on ice for 30 min under stirring, followed by centrifugation at 10 000*g* for 30 min at 4 °C. The supernatant was pooled together with the sucrose fraction and filtered through a 0.22 μm PES membrane (VWR) and used immediately or stored at 4 °C for further purification steps.

For perdeuteration in *E. coli*, we followed the protocol published by Sørensen et al.^22^ with slight variations. In brief, BL21 Star (DE3) cells transformed with the GbpA-encoding pET-26b(+) vector were grown for 5 h at 37 °C, 220 rpm, in 2.5 mL LB medium containing 50 μg/mL Kan, to an OD_600_ of 3. A 200 μL aliquot of this hydrogenated preculture was diluted in 2.5 mL of LB medium prepared with deuterium oxide as solvent (1/12.5 dilution) and grown for 6.5 h at 37 °C, 220 rpm, in the presence of 50 μg/mL Kan, to OD_600_ ≈ 2.5. The deuterated preculture was then transferred to 25 mL (1/10 dilution) deuterated M9glyc+ minimal medium and grown for 14 h (to OD_600_ ≈ 4) at 37 °C, 130 rpm, in the presence of 50 μg/mL Kan. Deuterated M9glyc+ media was produced following the recipe in Table S1, using anhydrous salts and deuterium oxide as solvent. Only the MEM vitamin and trace element solutions contained water as solvent. The antibiotics solution was also prepared using deuterium oxide as solvent. The main culture was started by adding 225 mL of deuterated M9glyc+ medium to the culture (1/10 dilution) and adjusting the concentration of antibiotic back to 50 μg/mL Kan. IPTG was added to a final concentration of 1 mM to induce expression after 9.5 h, when OD_600_ reached 2. After incubation for 20 h at 20 °C, 130 rpm, cells were harvested and subjected to periplasmic extraction as described above for the hydrogenated protein, with all the centrifugation steps performed at 4000*g* instead of 10 000*g*.

#### Expression of cctx2

C41 (DE3) cells (Sigma) transformed with pET-24b(+) CCTX2 vector were grown overnight at 30 °C, 120 rpm, in 50 mL LB medium^20^ with 100 μg/mL ampicillin (Amp). The main culture (1 L LB medium with 100 μg/mL Amp) was inoculated with the preculture to a 1/200 dilution factor and grown at 37 °C, 110 rpm. When OD_600_ reached 0.6, the culture was cooled down in an ice bath for 10–15 min, and 0.5 mM IPTG was added to induce expression. After incubation for 20 h at 20 °C, 110 rpm, cells were harvested at 4500*g*, 4 °C, for 30 min. The cells were used immediately or stored at −80 °C until use.

### Recombinant Expression in *Vibrio natriegens*

Most cultures of Vmax cells were grown in LB-v2 salts medium.

#### Expression of ctxAB and eltAB

For CT and LT production, 10 mL of LB-v2 salts media containing 25 μg/mL CAM was inoculated with cells harboring the pARCT5 or pARpLT5 vectors and grown at 30 °C and 180 rpm for 16 h. The cultures were diluted 1/100 in 500 mL of LB-v2 salts medium (25 μg/mL CAM) until OD_600_ reached ≈ 0.8 before induction with 0.2% l-arabinose (Sigma) at 30 °C, 140 rpm, for 20–22 h. CT and LT were harvested from the culture media by two rounds of centrifugation at 8500*g* for 30 min at 4 °C.

#### Expression of gbpA

GbpA was produced in minimal medium adapted for the growth of Vmax (which we refer to as M9max) in baffled flasks (for media recipe, see Table S2). All media contained 200 μg/mL Kan, and incubation was carried out at 30 °C and 120 rpm. Briefly, a preculture in 2 mL of LB-v2 salts medium was started from a glycerol stock and incubated for 5 h to OD_600_ ≈ 4. A growth culture of 10 mL of minimal medium with 200 μg/mL Kan was then inoculated with 100 μL of the preculture (1/100 dilution), allowing it to grow for 14 h to OD_600_ ≈ 9 before topping it up with 90 mL of minimal medium. After 3 h, *gbpA* expression was induced with the addition of 1 mM IPTG for 22 h, and the protein was harvested from the culture media by two rounds of centrifugation at 8500*g* for 30 min at 4 °C.

For perdeuteration in *V. natriegens*, we used the same recombinant construct and adapted the protocol described above. All media contained 200 μg/mL Kan, and incubation was carried out at 30 °C. Briefly, a preculture in 1 mL of LB-v2 salts medium was started from a glycerol stock and incubated for 3 h (until OD_600_ ≈ 2.5). Thereafter, 200 μL of this hydrogenated preculture was diluted in 2 mL of LB-v2 salts medium prepared with deuterium oxide as solvent (1/10 dilution) and grown for 4 h (to OD_600_ ≈ 2) at 37 °C, 220 rpm. The deuterated preculture was subsequently transferred to 10 mL (1/5 dilution) deuterated M9max minimal medium and grown for 14 h at 110 rpm, until it reached OD_600_ ≈ 4.5. Deuterated M9max minimal medium was produced following the recipe in Table S2, using anhydrous salts and deuterium oxide as solvent. Only the MEM vitamin and trace element solutions contained water as a solvent; the solution containing the antibiotic was prepared using deuterium oxide as solvent. The main culture was started by adding 90 mL of deuterated M9max medium to the culture (1/10 dilution). IPTG was added to a final concentration of 1 mM to induce expression after 6 h. After incubation for 20 h at 120 rpm and 30 °C, the culture media was harvested as described above by two rounds of centrifugation at 8500*g* for 30 min at 4 °C.

#### Expression of cctx2

For CCTX2, Vmax cells transformed with pET-24b(+) containing *cctx2* wild-type were grown at 30 °C, 120 rpm, overnight in the presence of 400 μg/mL Kan. 1 L of LB-v2 salts medium (400 μg/mL Kan) was inoculated with 1/200 preculture and incubated at 30 °C, 110 rpm. When the culture reached OD_600_ ≈ 0.8, expression was induced with 0.5 mM IPTG, and the culture was incubated at 30 °C, 110 rpm for 18 h. Because CCTX2 is not secreted, cells were harvested at 8500*g* for 30 min at 4 °C, and the pellet was stored at −80 °C until use.

### Protein Purification

#### Purification of CT and LT

All steps were carried out at 4 °C. CT was captured from the periplasmic fraction^24^ (*E. coli* expression) or the growth medium (Vmax expression) by immobilized metal affinity chromatography (IMAC), as the toxin carries two histidine residues that confer natural weak affinity for Ni^2+^ and Co^2+^.^25^ The filtered solutions were directly applied onto a HiTrap Talon crude 5 mL column (Cytiva) previously equilibrated with Talon A buffer, followed by a 15 column-volume (CV) wash with the same buffer and elution with 10 CV Talon B (50 mM sodium phosphate pH 8.0, 300 mM NaCl, 50 mM imidazole). The protein was concentrated by ultrafiltration (4 °C, 3500*g*) using Amicon Ultra Centrifugal Filter Units 10K molecular weight cutoff (MWCO) (Merck). CT produced in *E. coli* was subsequently dialyzed into IEX A buffer (50 mM Tris-HCl pH 8.0) and purified by cation-exchange chromatography with a HiTrap SP (GE Healthcare) column. CT holotoxin eluted in the flow-through, while free CTB pentamers were eluted with a 5 CV 0–100% linear gradient of IEX A to IEX B buffer (50 mM Tris-HCl pH 8.0, 1 M NaCl). The holotoxin-containing flow-through was concentrated and further purified by size-exclusion chromatography (SEC) with a Superdex 200 16/60 GL or Superdex 200 increase 10/300 GL column (Cytiva) equilibrated with phosphate-buffered saline, pH 7.4 (PBS). For CT produced in Vmax, the cation-exchange step was not needed. Fractions containing pure protein were pooled, concentrated by ultrafiltration as described above, and stored at 4 °C.

As an alternative to IMAC, CT was captured from the medium by galactose-affinity chromatography, exploiting the protein’s affinity for sugars. This was the primary method to capture LT from the medium, as it does not contain the histidine residues that confer affinity for divalent metals. Briefly, the filtered supernatant was loaded onto 3–4 mL immobilized d-galactose gel (Pierce, Thermo Scientific) equilibrated by gravity flow with Gal A buffer (50 mM Na-phosphate pH 7.4, 200 mM NaCl). After a 15 CV washing step with the same buffer, the protein was eluted with 10 CV of Gal B buffer (50 mM Na-phosphate pH 7.4, 200 mM NaCl, 300 mM d-galactose), concentrated by ultrafiltration and further purified by SEC in the same way as CT, using a Superdex 200 increase 10/300 GL column (Cytiva).

#### Purification of GbpA

To capture GbpA from the culture media of Vmax, the media were first dialyzed overnight at 4 °C against 20 mM Tris-HCl pH 8.0, 100 mM NaCl (volume ratio 1:20 sample:buffer) using 10K MWCO SnakeSkin dialysis tubing (ThermoScientific). Dialyzed supernatant was subsequently loaded onto an equilibrated 5 mL HiTrap Q XL column (Cytiva) for anion-exchange chromatography (AEX). For the protein produced in *E. coli*, the fractions resulting from the osmotic shock were directly loaded onto the AEX column. After a washing step with 20 CV binding buffer, the protein was eluted over a 12 CV 0–100% linear gradient with elution buffer (20 mM Tris-HCl pH 8.0, 400 mM NaCl). Fractions were analyzed by SDS-PAGE and those containing the target protein were pooled and concentrated with Amicon Ultra Centrifugal Filter Units 10K MWCO (Merck). GbpA was further purified by SEC using a Superdex 200 Increase 30/100 GL column (Cytiva) equilibrated with 20 mM Tris-HCl pH 8.0, 100 mM NaCl. For perdeuterated GbpA, a Superdex 75 Increase 30/100 GL column (Cytiva) equilibrated in the same buffer was used instead.

#### Purification of CCTX2

CCTX2 is retained in the cytoplasm of the cells. Cell pellets were resuspended in 5 mL lysis buffer (50 mM Na-HEPES, 200 mM NaCl, 2 mM EDTA, pH 7.5, supplemented with 5 mM dithiothreitol (DTT) and 1x *cO*mplete protease inhibitor cocktail (Roche)) per gram of wet cell paste and incubated for 1 h at 4 °C under stirring. The suspension was sonicated for 1 min (20% amplitude, 3 s on/7 s off) for *E. coli* cells and 5 min (30% amplitude, 3 s on/7 s off) for *V. natriegens* cells. The lysate was clarified at 40 000*g*, 4 °C for 30 min to remove cell debris. The resulting supernatant containing soluble CCTX2 was then filtered through a 0.22 μm PES membrane filter and loaded onto a 5 mL Tricorn column packed with immobilized d-galactose gel (Pierce, Thermo Scientific) previously equilibrated with 6 CV loading buffer (20 mM Na-HEPES, 500 mM NaCl, pH 7.5). After a wash with 6 CV loading buffer, CCTX2 was eluted with 10 CV elution buffer (20 mM Na-HEPES, 500 mM NaCl, 1 M d-galactose, pH 7.5). The fractions containing CCTX2 were pooled and concentrated with an Amicon Ultra Centrifugal Filter Units 30K MWCO (Merck). Finally, CCTX2 was purified by SEC using a Superdex 200 Increase 30/100 GL column equilibrated with 50 mM Na-HEPES, 150 mM NaCl, 100 mM d-galactose, pH 7.5. In the case of the *E. coli* expression system, the whole fraction was loaded, whereas only half of the sample from the Vmax expression system was used.

### Characterization of Proteins and Functional Analysis

#### Crystallization of CT, X-ray Data Collection, and Refinement

Purified CT was dialyzed using a Pur-A-Lyzer Midi 3,500 MWCO (Sigma) into buffer G (50 mM Tris pH 7.4, 200 mM NaCl, 1 mM EDTA, 3 mM NaN_3_). The protein was crystallized by the sitting-drop vapor-diffusion method. Crystallization experiments were set up on 2-Lens UVXPO plates (SwissCI) using a crystallization robot (Oryx 4, Douglas Instruments) at 20 °C. Crystals grew from 2 μL drops containing CT (5.76 mg/mL) and reservoir solution (0.125 M magnesium acetate, 24% w/v PEG 3350, and 300 mM d-galactose in Buffer G), mixed in a 1:1 volume ratio. Crystals were harvested with a nylon loop and cryo-protected in reservoir solution complemented with glycerol to a final concentration of 20% v/v. Cryo-protected crystals were flash-cooled in liquid nitrogen and shipped in a cryo-cooled dewar to the European Synchrotron Radiation Facility (ESRF) for data collection. Data were collected at ID30B, ESRF, Grenoble (France). Diffraction images were integrated and scaled using *autoPROC*;^26^ integrated and scaled intensities were merged and truncated with *AIMLESS*(27,28) from the *CCP*4 software suite.^29^ The resolution cutoff was chosen based on the CC_1/2_, which, as described by Karplus & Diederichs,^30^ could be as low as 0.1 and still provide structural information. The structure was solved by molecular replacement using a previous CT structure determined to 1.9 Å (PDB ID:1S5E)^31^ as search model with *Phaser*(32) (*CCP*4 suite).^29^ Refinement was performed by alternating cycles of manual rebuilding using *Coot*(33) and maximum-likelihood refinement with *REFMAC*5.^34^ Occupancy refinement was carried out with *phenix.refine* from the *Phenix* software suite.^35^ Data collection and refinement statistics are summarized in Table S3. The structure was deposited in the Protein Data Bank (PDB),^36^ with accession ID 8QRE.

#### Toxicity Assays

CHO cells seeded to a 24-well plate and grown overnight to 80% confluency were incubated for 2 h in serum-free medium containing 10-fold serial dilutions of CT, either purchased from Sigma (catalog #227036) or produced in Vmax. Cells were lysed in sample diluent from the Arbor Assays cAMP Direct ELISA, with clarified supernatants applied directly to the ELISA plate and processed for cAMP levels according to the manufacturer’s instructions. The cAMP content present in unintoxicated control cells was background-subtracted from cAMP values recorded for the toxin-treated cells. The cAMP responses were then standardized to the maximal signal obtained with the commercial toxin by dividing the value from each technical replicate by the average cAMP response generated from cells exposed to 100 ng/mL of Sigma CT.

#### Quantitation of Protein Deuteration

The buffer of GbpA samples was exchanged to pure water (hydrogenated) by concentration and subsequent dilution using Vivaspin 20 centrifugal filters (Sartorius). The protein was stable in water and the samples were concentrated to 0.3–0.6 mg/mL. Electrospray ionization time-of-flight (ESI-TOF) mass spectrometry analysis was used for mass determination, using direct injection into a maXis II ETD HRMS QTOF machine (Bruker). Spectra containing peaks for differently charged species were obtained and deconvoluted to a single peak for the +1 species using the charge deconvolution tool for proteins from the Compass data analysis software (Bruker). The theoretical and experimental values for the molecular mass of GbpA are shown in Table S4. Calculation of deuteration level for nonlabile hydrogens was performed as described by Meilleur et al.^37^ using eq 1:
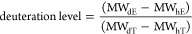
1where MW_dE_ and MW_hE_ correspond to the experimentally determined masses of the deuterated and nondeuterated proteins, respectively, and MW_dT_ and MW_hT_ correspond to the theoretical masses of both species.

## Results and Discussion

### Production of Recombinant Cholera Toxin

**Figure 1:**
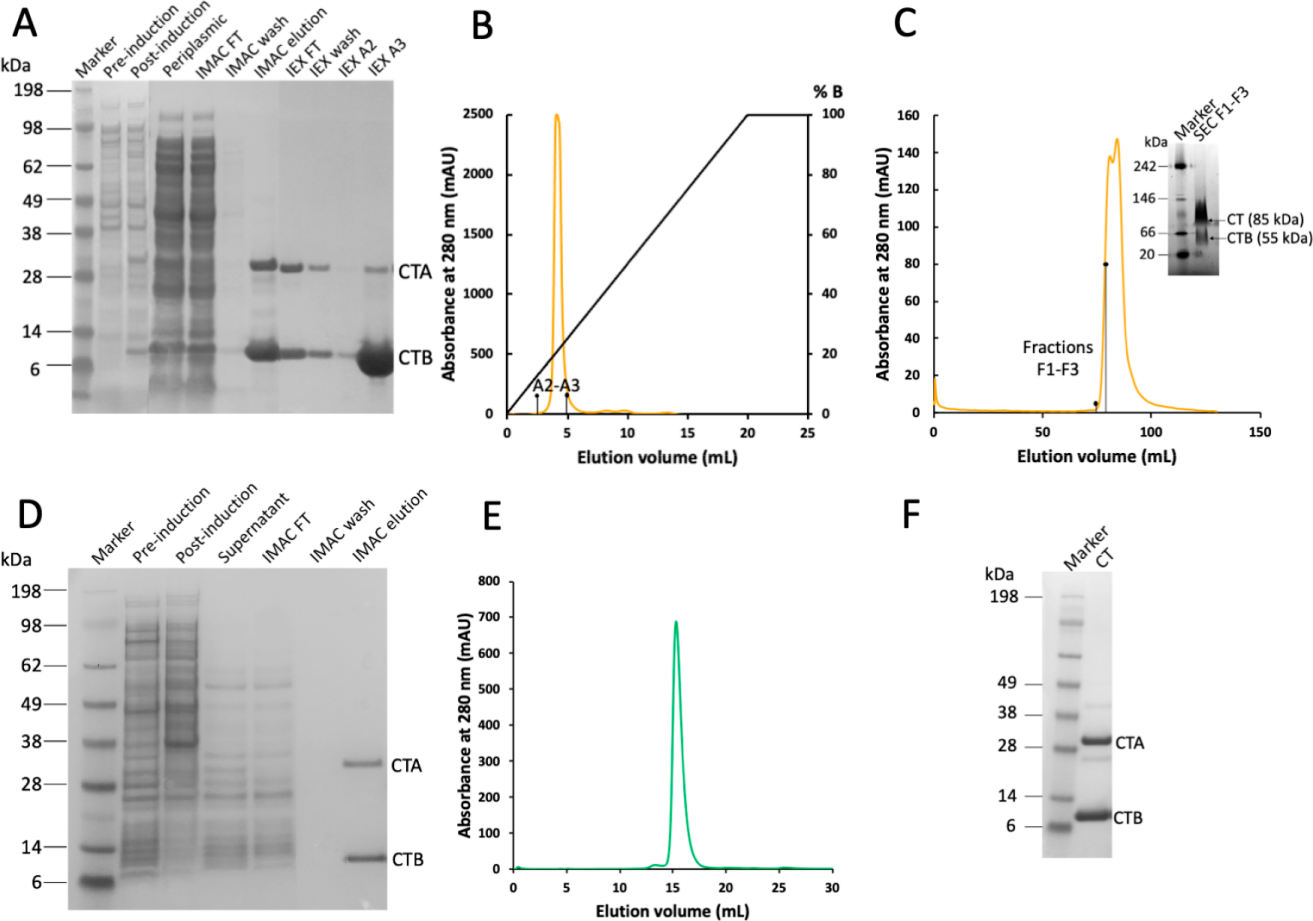
CT production in *E. coli* and Vmax. (A) SDS-PAGE analysis of CT samples obtained by expression in *E. coli* and purification by IMAC and ion-exchange chromatography. Periplasmic: periplasmic fraction, FT: flow-through. (B) Cation-exchange chromatogram of CT produced in *E. coli*. At pH 8.0, the holotoxin is negatively charged and elutes in the flow-through and wash fractions, while (most) CTB binds to the column and elutes with a linear gradient of buffer B. (C) SEC chromatogram of CT produced in *E. coli*. SEC was performed on the holotoxin-containing flow-through and wash from the IEX step. SEC fractions F1–F3 (first half of peak in C) on native PAGE (inset) still contained considerable amounts of CTB in the purified holotoxin sample. (D) SDS-PAGE gel from CT expression in Vmax, where CT is secreted into the growth medium. (E) SEC chromatogram of CT produced in Vmax, and (F) SDS-PAGE gel of SEC peak (compared to the molecular mass marker).

In our lab, we most commonly use *E. coli* as expression host. For producing CT, our optimized protocol involved purification from the bacterial periplasmic space by IMAC, followed by ion-exchange and size-exclusion chromatography (Figure 1A–C). This protocol yielded only 0.2 mg of pure protein per L culture media, which was insufficient for our planned structural studies without significant scale-up. One of the main issues with this protocol was the presence of excess CTB compared to the CT holotoxin, which proved difficult to separate by chromatographic techniques (Figure 1A,C).

As an alternative, we decided to test expression in Vmax, an engineered strain derived from the nonpathogenic bacterium *Vibrio natriegens*.^6^ Since this bacterium is more closely related to *V. cholerae* than *E. coli*, we hoped to obtain higher yields with this expression host. To explore expression, we transformed Vmax cells with the same plasmid used for *ctxAB* expression in *E. coli*. This plasmid contains several elements compatible with Vmax, including the p15A origin of replication and *araBAD* promoter.^18,38^

In our initial test, expression was induced with 0.2% l-arabinose—the same concentration used for induction in *E. coli*—when the cells reached OD_600_ ≈ 0.8. Due to the fast growth of Vmax, such density was achieved just 2 h after inoculation. To evaluate if the protein was produced, samples before and 20 h after induction were collected and analyzed by SDS-PAGE (Figure 1D, first two lanes after marker). In addition, we collected a postinduction sample of the culture supernatant (Figure 1D, lane 3 after marker), since we anticipated that CT could be secreted by the Vmax T2SS, the same machinery used by *V. cholerae* to secrete native CT. However, CT was not visible on the gel (Figure 1D), neither in cell samples (pre or postinduction) nor in the culture supernatant. This indicated that the protein was either too diluted to be clearly seen in the supernatant fraction or that it was not produced. To test if CT was secreted, the supernatant was directly loaded onto an IMAC column, and the elution fraction was analyzed by SDS-PAGE. Two clear bands matching the sizes of CTA and CTB were observed (Figure 1D, last lane), confirming successful production of the toxin. CT appeared to already be almost pure after this step; nevertheless, the protein was subjected to SEC to assess the presence of CTB and remove aggregates or contaminants that may not be visible on the gel. A single sharp peak was obtained (Figure 1E, with corresponding SDS-PAGE gel in Figure 1F), suggesting that there was little or no contamination by CTB, a major issue for expression in *E. coli* (Figure 1 C).

For each of the independent trials of toxin production in Vmax, at least 10 times more CT was obtained in Vmax compared to *E. coli* (2–12 mg per L culture in Vmax vs. 0.2 mg in *E. coli*; Table 2). By inducing expression with 0.02% l-arabinose and loading the medium on a galactose resin instead of IMAC, up to 20% higher yields were obtained. This protocol could be further optimized, but in its current form, it already allowed us to obtain sufficient amounts of toxin for structural studies. Furthermore, secretion of the protein to the growth medium simplified the downstream purification procedure and made it more time-efficient. The use of Vmax thus allowed us to increase the yield and purity of CT, which is essential for structural studies.

**Figure 2:**
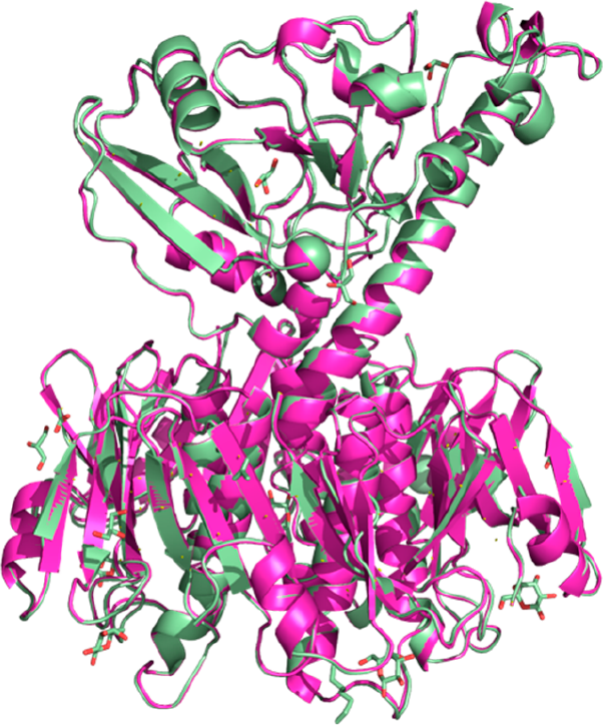
Comparison of CT crystal structures. CT produced in Vmax (light green; PDB ID: 8QRE, this work), superimposed onto the published crystal structure of CT produced in *E. coli* (magenta; PDB ID: 1S5E).^31^ RMSD = 0.3 Å.

To verify that the CT holotoxin produced in Vmax was correctly folded, we determined its crystal structure to 2.3 Å resolution (Figure 2, PDB ID: 8QRE). The structure was refined to *R*/*R*_free_ values of 22.1/26.2% and exhibited good geometry based on the Ramachandran plot as well as small deviations from ideal bond lengths and angles (Table S3). The crystal structure is essentially identical to the CT crystal structure published earlier by the Hol lab (Figure 2, PDB ID: 1S5E),^31^ from protein produced in *E. coli*, with an RMSD value for all C_α_ atoms of 0.3 Å confirming the correct folding of the holotoxin.

**Table 2:** Comparison of Yields Obtained with *E. coli* and Vmax Expression Systems

Protein	Yield in *E. coli* (no.)^a^	Yield in Vmax (no.)^a^
CT	0.2 mg/L (>3)	2–12 mg/L (>3)
LT	0.1 mg/L (3)^b^	7 mg/L (2)^c^
^1^H-GbpA	15 mg/L (>3)	90 mg/L (>3)
^2^H-GbpA	15 mg/L (>3)	42 mg/L (2)
CCTX2	0.4 mg/L (2)	10 mg/L (1)

a Yields are expressed in mg of protein per L bacterial culture, with the number of replicates given in parentheses.

b Yield for hLT (LT variant infecting humans).

c Yield for pLT (LT variant infecting pigs).

**Figure 3:**
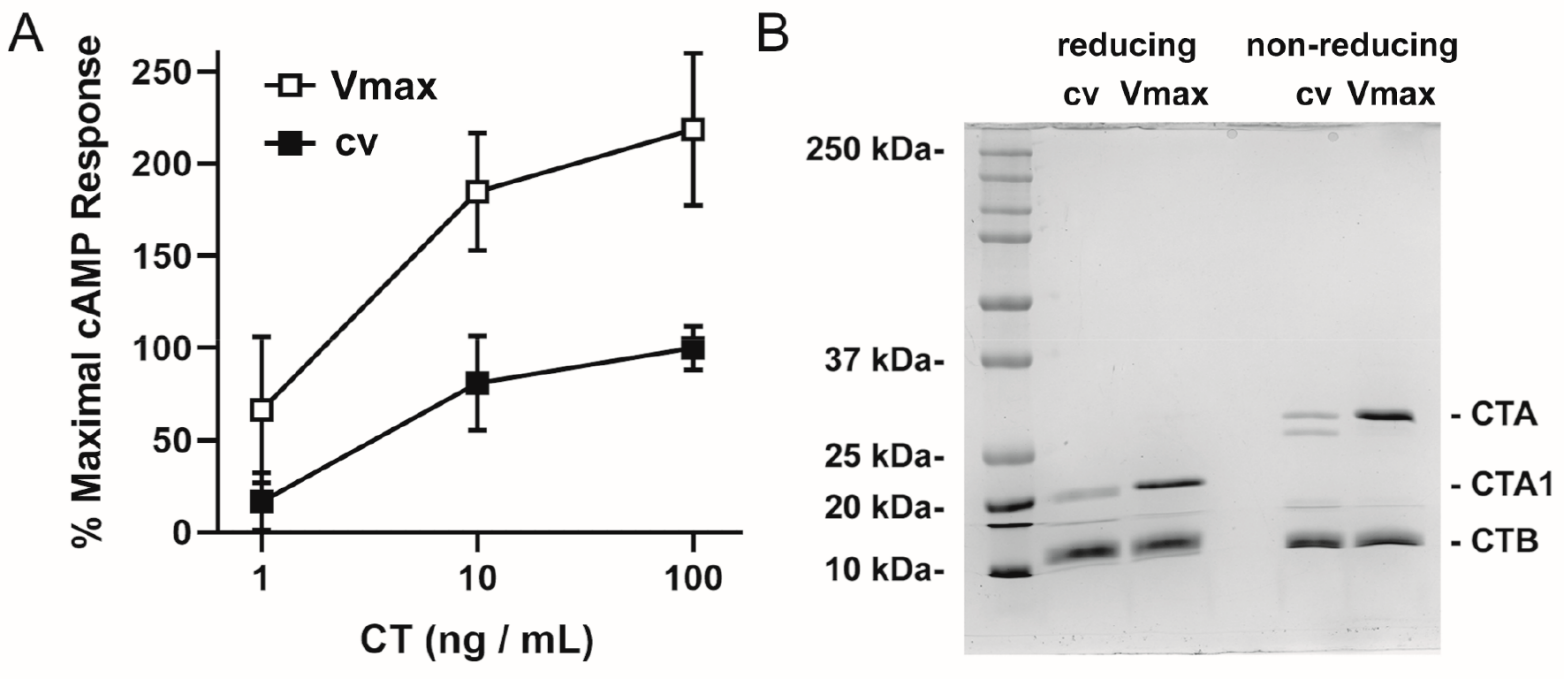
Toxicity and stoichiometry for commercial and Vmax preparations of CT. (A) CHO cells were incubated for 2 h with 10-fold dilutions of CT purchased from a commercial vendor (cv, filled squares) or purified from Vmax (Vmax, open squares). An ELISA was then used to quantify cAMP levels from the intoxicated cells. Background-subtracted data were expressed as percentages of the response elicited from cells challenged with 100 ng/mL of the commercial toxin and represent the means ± standard deviations of nine technical replicates from three independent experiments. (B) Samples of CT purchased from a commercial vendor (cv) or produced in Vmax (Vmax) were resolved by SDS-PAGE under reducing and nonreducing conditions. The samples (4 μg per lane) were visualized by Coomassie stain. The entire gel is shown, with the molecular mass marker from select protein standards shown on the left.

We also confirmed that the produced toxin retains its biological activity. In fact, the toxin produced in our lab elicited a stronger cAMP response in intoxicated cells than CT obtained from a commercial supplier (Figure 3A). This difference likely relates to the greater quantity of intact holotoxin in the Vmax preparation when compared to the commercial preparation: both preparations had equivalent levels of the CTB subunit as assessed by SDS-PAGE, but the commercial preparation had a lower quantity of CTA and, thus, less functional toxin (Figure 3B). Additionally, commercial CT is delivered as a lyophilized sample, increasing the risk of denaturation.

Our study is not the first to show that CT holotoxin (or ‘*choleragen*’) production simultaneously yields CTB (also referred to as ‘*choleragenoid*’).^39^ Characterization of the subunit assembly process, mainly based on studies of the CT ortholog LT from *E. coli*,^40−42^ confirmed that B-pentamers can form and be exported on their own, whereas uncomplexed A-subunits remain associated with the cells.^40^ Interestingly, assembly into holotoxins was shown to be promoted by the A-subunit,^41,42^ which on its own is highly unstable and prone to degradation. Since expression in Vmax was performed at a lower temperature compared to *E. coli* (30 versus 37 °C in *E. coli*), we suspect that CTA may be less affected by thermal degradation in this system, leading to a more homogeneous production of the holotoxins.

Previous approaches to achieve mg amounts of CT involved the use of toxigenic *V. cholerae* strains.^39,40,43−45^ However, these strains are inherently pathogenic and are limited to production of the native toxin. Also nontoxigenic *V. cholerae* strains have been used for producing recombinant CTB;^46−48^ however, transforming such strains with the desired plasmids proved challenging.^47^ To the best of our knowledge, these strains have not been used for producing CT. Using Vmax for CT production thus has advantages over other *Vibrio* strains, both regarding safety of handling and breadth of applications.

In summary, with Vmax we could increase the yield and purity of CT, which is crucial for structural studies.

### Production of Recombinant Heat-Labile Enterotoxin

**Figure 4:**
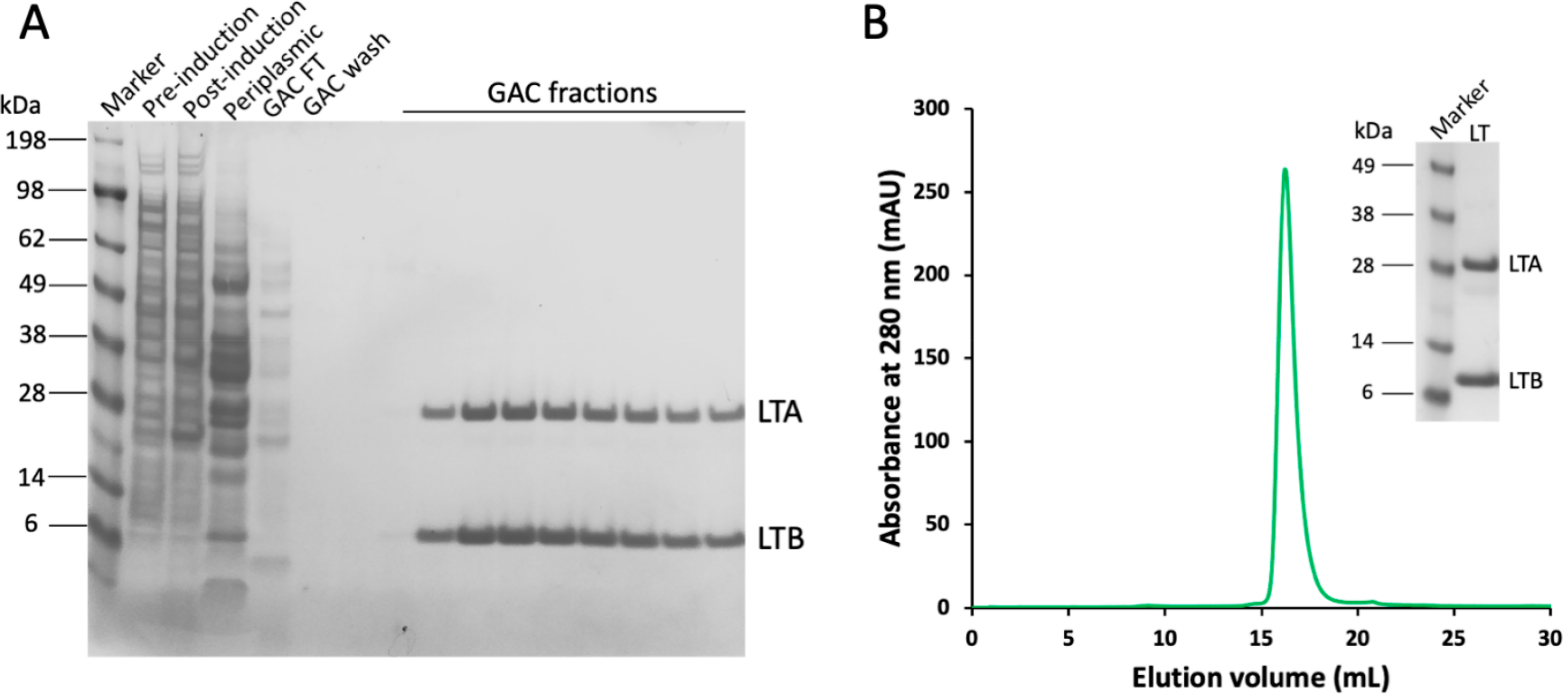
LT production in Vmax. (A) SDS-PAGE analysis of LT samples obtained for expression in Vmax and purification from the culture supernatant by galactose-affinity chromatography (GAC). (B) SEC chromatogram of LT captured by GAC, and SDS-PAGE of SEC fractions (inset).

A close homologue of CT is LT from ETEC.^49^ Our promising results using Vmax for expression of *ctxAB* encouraged us to test this host for the production of pLT, the toxin originating from ETEC strains infecting pigs. Since CT and LT share more than 80% sequence identity,^49^ we applied the same expression protocol developed for CT to LT production. Like CT, LT was secreted into the medium as an intact holotoxin. However, LT was purified by galactose-affinity chromatography (Figure 4A), as it lacks the histidine residues that confer natural affinity for divalent metals in CT.^25^ After SEC, no free CTB was present in the toxin preparation (Figure 4B). Yields of purified LT (7 mg of protein per L medium) were comparable to those obtained for CT (Table 2). This stands in contrast to our previous experience using *E. coli* to purify hLT (the toxin originating from ETEC strains infecting humans): three rounds of expression were required to produce just 1 mg of toxin from 12 L expression media (unpublished data; reported in Table 2). Yields of both CT and LT were thus substantially higher in Vmax than for *E. coli*.

CT and LT production in Vmax has an additional advantage over their expression in *E. coli*: the toxins are secreted as soluble proteins from Vmax but not *E. coli*. Both CT and LT can be exported across the *E. coli* outer membrane by its T2SS, yet the toxins remain associated with the *E. coli* surface via binding to the outer-membrane lipopolysaccharides (LPS).^50,51^ CT and LT do not have affinity for *Vibrio* LPS,^51^ which explains why they are secreted as soluble proteins from *Vibrio* strains.

### Production of Another *V. cholerae* Protein: GbpA

**Figure 5:**
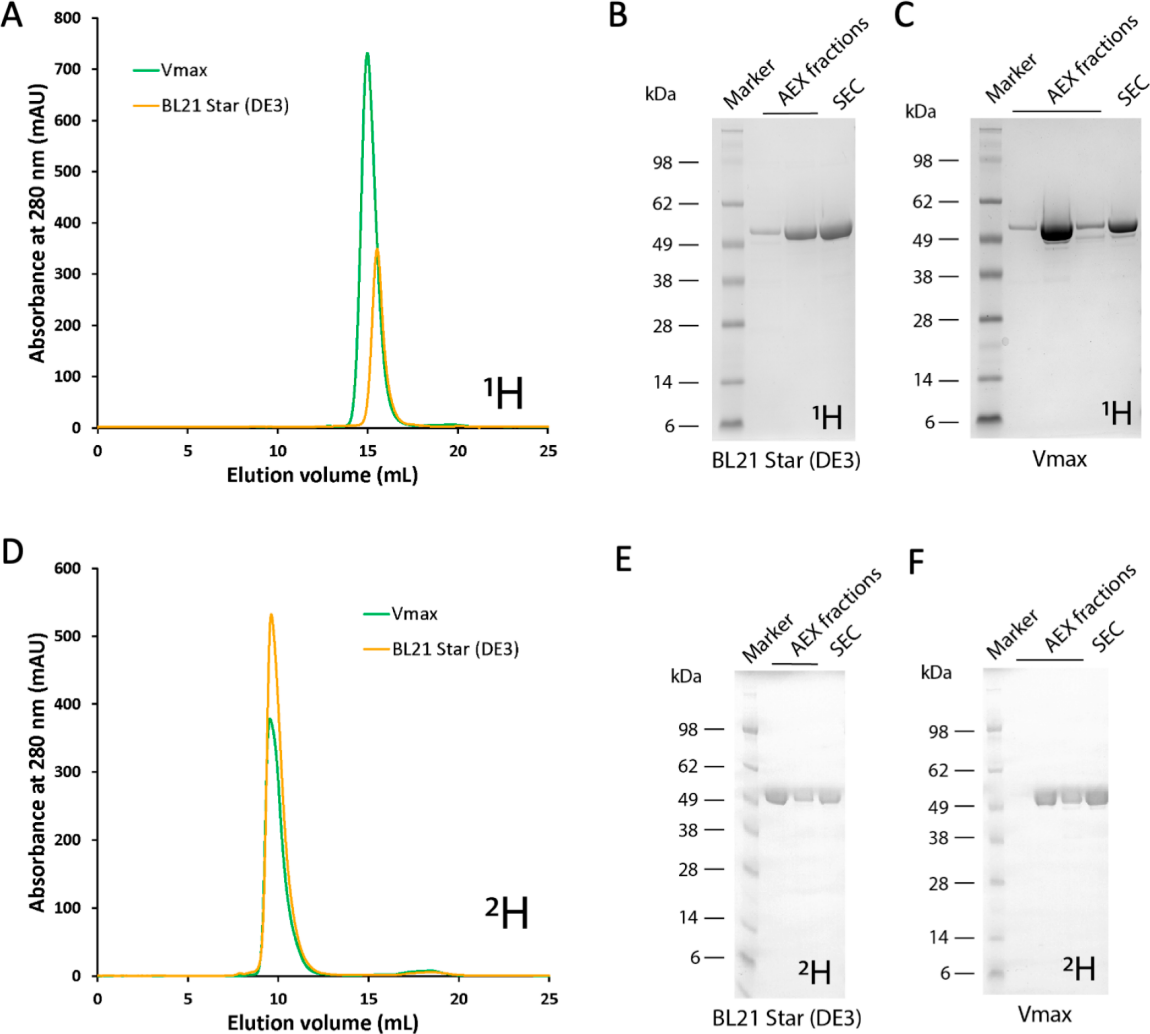
GbpA production in *E. coli* and Vmax. (A) SEC chromatogram (Superdex 200 Increase 30/100 GL column) of hydrogenated (^1^H) GbpA expressed in *E. coli* BL21 Star(DE3) (light orange) and in Vmax (green). Several SEC runs were performed for each purification batch; therefore, the intensity of absorbance is not correlated with yield. (B,C) SDS-PAGE analysis of GbpA hydrogenated (^1^H) samples expressed in Vmax or *E. coli* BL21 Star(DE3) after the AEX and SEC purification steps. (D) SEC chromatogram (Superdex 75 Increase 30/100 GL column) of perdeuterated (^2^H) GbpA expressed in *E. coli* BL21 Star(DE3) (light orange) and in Vmax (green). Again, SEC was performed in several injections per batch, and the amounts shown do not correlate with yield. (E,F) SDS-PAGE analysis of GbpA perdeuterated (^2^H) samples expressed in Vmax or *E. coli* BL21 Star(DE3) after the AEX and SEC purification steps. Deuteration levels of the produced protein were determined to be 96 and 97% for the protein produced in *E. coli* and Vmax, respectively. ^1^H refers to “common” hydrogen (also called protium); ^2^H refers to deuterium.

Another protein that is natively secreted by the T2SS is the *V. cholerae* colonization factor GbpA. This adhesin binds to chitin and mucins, helping the pathogen to survive in its natural marine environment and to colonize the human intestine, respectively.^52^ In our lab, GbpA has routinely been produced in *E. coli*, in both TB and minimal media,^22^ and purified from the *E. coli* periplasmic fraction by ion-exchange chromatography and SEC. Interestingly, we found in previous work that better yields were obtained when producing the protein in minimal media instead of the nutrient-rich media TB (15 mg per L in M9 vs 7 mg per L in TB).^22^ We suspected that this might be due to misfolding of GbpA when produced too quickly in TB, whereas slower expression in M9 would result in less aggregates. Although GbpA was already produced in relatively high yields in *E. coli*, we decided to test expression in Vmax, given our encouraging results with the bacterial toxins (Figures 1 and 4). When expressed in Vmax, GbpA could be seen in the culture supernatant (Figure S2), indicating that the protein is recognized by the T2SS machinery of *V. natriegens* and secreted. GbpA is produced in much greater amounts than CT, which is not surprising since *gbpA* is also highly expressed in *E. coli*. Production in Vmax not only increased GbpA yield by more than 6-fold compared to production in *E. coli* but also simplified protein purification. SEC chromatograms and SDS-PAGE analysis of the produced samples are shown in Figure 5A–C. Similar results were recently reported in an independent study by Schwarz et al., who used GbpA as a secretion and affinity purification tag for an antimicrobial peptide produced in *V. natriegens*.^12^ Because GbpA is isolated from the culture supernatant, contaminants from the periplasm are avoided, and aggregation is less likely to occur. These results demonstrate that Vmax is a suitable host to express virulence factors secreted by the T2SS, both from *V. cholerae* and other bacteria like ETEC.

Since we intend to perform neutron scattering experiments with GbpA,^22^ we would strongly benefit from producing perdeuterated protein. However, this is not necessarily easy to achieve and also quite expensive. Given our encouraging results using *V. natriegens* for producing hydrogenated GbpA and our previous expertise producing perdeuterated GbpA in *E. coli*,^22^ we tested *gbpA* expression with the Vmax system, using deuterated minimal media and deuterium oxide as solvent. Following the study by Cai et al.^23^ and our own work,^22^ we used deuterated glycerol as a carbon source, given its significantly lower price compared to deuterated glucose. To our knowledge, isotopic labeling using Vmax has only been reported for ^15^N^13^ but never been tested for protein deuteration. Indeed, we were able to produce deuterated GbpA using Vmax in almost 3-fold higher yields than in *E. coli* (41.6 mg per L in Vmax vs 14.8 mg per L in *E. coli*). Moreover, downstream processing of the produced protein was simplified, as was the case for hydrogenated media. SEC chromatograms and SDS-PAGE analysis of the deuterated samples are shown in Figure 5D–F. The protein deuteration level in Vmax was quantified to be 97% by mass spectrometry and is thus comparable to deuteration in *E. coli* BL21 Star (DE3) (96%; Table S4).

Interestingly, the molecular mass for GbpA produced in Vmax is not identical to that produced in *E. coli*, with both deuterated and nondeuterated proteins produced in *Vibrios* exhibiting an increased mass by approximately 500 Da (i.e., 498 and 520 Da for the nondeuterated and deuterated species, respectively). This mass difference could correspond to previously unidentified native O-glycosylation by *V. natriegens*, mimicking the glycosylation pathways of *V. cholerae*.^53^ With O-glycosylation known to be important for *V. cholerae*, including processes in which GbpA has been implicated, such as chitin utilization and biofilm formation,^53^ this may open the door to the study of natively modified protein targets. Clearly, this putative glycosylation needs to be further studied.

### Production of a Nonsecreted Fungal Protein Toxin: CCTX2

**Figure 6:**
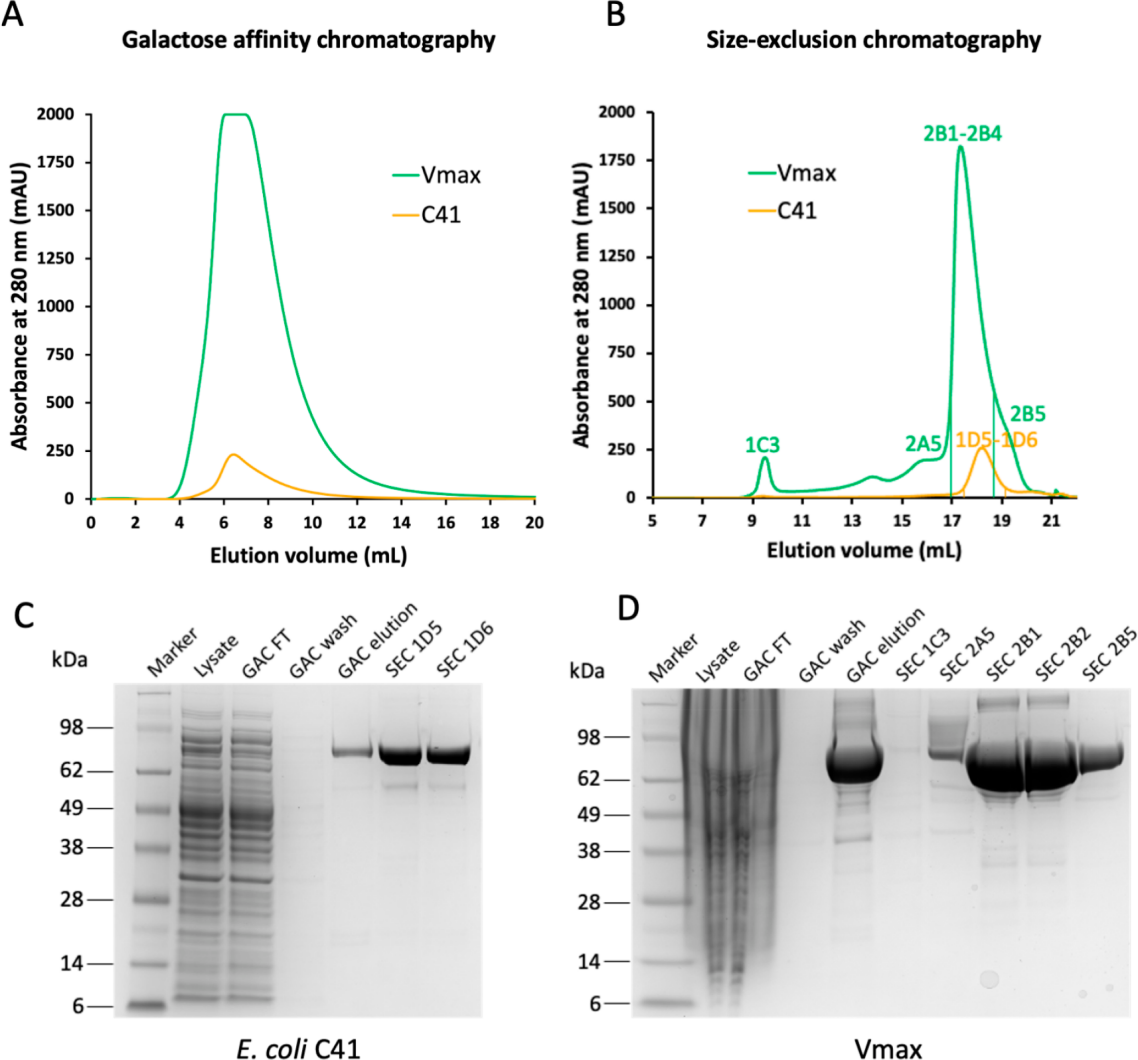
CCTX2 production in *E. coli* and Vmax. (A) Galactose-affinity chromatography of CCTX2 produced in 1 L cultures from *E. coli* (light orange) and *V. natriegens* (green). (B) Matching SEC chromatogram comparing production in *E. coli* (light orange) and *V. natriegens* (green) for 0.5 L cultures. (C,D) SDS-PAGE of CCTX2 samples from expression in *E. coli* C41(DE3) and Vmax, matching purification fractions labeled in panel B.

To test the full potential of the Vmax system, we applied our protocol to CCTX2, a fungal nematotoxin, for which no structural information is available to date and very little is known about its function. Preliminary work in both our own lab at UiO and that of our collaborators at ETH Zurich (Künzler lab) had yielded only low amounts of CCTX2 in *E. coli* (approximately 0.4 mg per 1 L culture media). Since structural characterization often requires large amounts of protein, this seemed to be a perfect test case. We carried out *cctx2* expression in Vmax and compared its yield in postinduction samples to *E. coli* C41(DE3), which was previously selected as the best-expressing strain from a large library of *E. coli* strains (unpublished). As seen in Figure 6, Vmax cells clearly produced a greater amount of protein than the *E. coli* system. Purification was performed using the same protocol for both strains, revealing the greater potential of Vmax already at the stage of galactose-affinity chromatography. The elution profile shows a very high amount of protein, with the UV_280_ detector reaching saturation for protein expressed in Vmax (Figure 6A). Purification by SEC on Superdex 200 Increase 30/100 GL resulted in large amounts of pure CCTX2, with higher *A*_280_ absorption intensity compared to C41(DE3) cells (Figure 6B). This was confirmed by SDS-PAGE (Figure 6C,D). After SEC, the sample from Vmax provided yields of approximately 10 mg of protein per liter culture (Table 2). The new protocol thus resulted in a 26-fold increase in yield.

## Conclusion

*V. natriegens* proved to be a highly advantageous host for high-yield production of virulence factors naturally secreted by the T2SS. In Vmax, the yield of CT was 10-fold higher when compared to *E. coli*, and a 6-fold increase was obtained for GbpA (3-fold increase for GbpA deuteration). Moreover, secretion of the recombinant proteins simplified the purification process and, in several cases, improved sample purity, since less contaminants were present in the culture supernatant compared to the bacterial cytosol and periplasm. For the fungal toxin CCTX2, a test case representing nonsecreted proteins, Vmax even allowed us to increase the production efficiency by 26-fold, which greatly benefits our ongoing structural characterization. Finally, *V. natriegens* proved to be an efficient tool for protein perdeuteration and may allow native-like glycosylation. We hope that our work encourages the use of *V. natriegens* as an alternative expression host for the production of other difficult expression targets, not only from *Vibrio* species but also from other bacteria and fungi. From this proof-of-principle study, we can conclude that *V. natriegens* is also useful for protein perdeuteration and for isotope labeling in general.

## Supporting Information Available

*Figure S1*. Plasmid maps; *Figure S2*. SDS-PAGE analysis of GbpA fractions before purification; *Table S1*. M9glyc+ minimal medium for GbpA production in BL21(DE3); *Table S2*. M9max minimal medium for GbpA production in Vmax; *Table S3*. Data collection and refinement statistics for CT produced from Vmax; *Table S4*. Quantification of GbpA deuteration (PDF)

## Accession Codes

UniProt IDs: P01555 and E9RIX3 (Genes *ctxA* and *ctxB*). PDB ID: 8QRE; UniProt IDs: P06717 and P32890 (Genes *eltA* and *eltB*); UniProt ID: Q9KLD5 (Gene *gbpA*); UniProt ID: A8NDT7 (Locus tag CC1G_10077, gene *cctx2*).

## Abbreviations

AEX: anion-exchange chromatography
CAM: chloramphenicol
CT: cholera toxin
CTA: catalytic A-subunit of CT
CTB: homopentamer of receptor-binding B-subunits of CT
CCTX2: *Coprinopsis cinerea* toxin 2
DTT: dithiothreitol
EDTA: ethylenediaminetetraacetic acid
ESRF: European Synchrotron Radiation Facility
ETEC: enterotoxigenic *Escherichia coli*
GAC: galactose-affinity chromatography
GbpA: *N*-acetylglucosamine-binding protein A
HEPES: N-(2-hydroxyethyl)piperazine-N′-(2-ethanesulfonic acid)
hLT: heat-labile enterotoxin from ETEC strains infecting humans
IMAC: ion-immobilized affinity chromatography
IPTG: isopropyl β-d-1-thiogalactopyranoside
Kan: kanamycin
LB: Luria–Bertani
LT: heat-labile enterotoxin
LTA: catalytic A-subunit of LT
LTB: homopentamer of receptor-binding B-subunits of LT
M9max: minimal medium adapted for the growth of Vmax X2 cells
MWCO: molecular weight cutoff
OD: optical density
PBS: phosphate-buffered saline pH 7.4
PMSF: phenylmethylsulfonyl fluoride
PES: polyethersulfone
pLT: heat-labile enterotoxin from ETEC strains of porcine origin
SDS-PAGE: sodium dodecyl sulfate–polyacrylamide gel electrophoresis
SEC: size-exclusion chromatography
TB: terrific broth
Tris: tris(hydroxymethyl)aminomethane
T2SS: Type II secretion system
Vmax: engineered strain of *Vibrio natriegens* commercialized as Vmax X2 (originally as Vmax Express)
