# Supplementary material for: Using Vibrio natriegens for High-Yield Production of Challenging Expression Targets and for Protein Perdeuteration
Source: Biochemistry. 2024 Feb 15;63(5):587–98. doi: 10.1021/acs.biochem.3c00612 (PMC10919088; doi:10.1021/acs.biochem.3c00612)
Supplement: Supplementary file 1 — bi3c00612_si_001.pdf [file bi3c00612_si_001.pdf]

SUPPORTING INFORMATION

Using *Vibrio natriegens* for high-yield production of challenging expression targets and for protein perdeuteration

Natalia Mojica[1], Flore Kersten[1,2#], Mateu Montserrat-Canals[1,2#], G. Robb Huhn III[3§], Abelone M. Tislevoll[1], Gabriele Cordara[1], Ken Teter[3], Ute Krengel[1*]

[1]Department of Chemistry, University of Oslo, NO-0315 Blindern, Oslo, Norway

[2]Centre for Molecular Medicine Norway, University of Oslo, NO-0318 Blindern, Oslo, Norway

[3]Burnett School of Biomedical Sciences, College of Medicine, University of Central Florida, Orlando, FL 32816, U.S.A.

[#]F.K. and M.M.-C. contributed equally to this work.

[§]Present address: G. Robb Huhn III, Biology Department, Stetson University, DeLand, Florida, U.S.A.

*corresponding author: ute.krengel@kjemi.uio.no

LIST OF MATERIAL INCLUDED:

Figures S1-S2 (S1, Plasmid maps; S2, SDS-PAGE analysis of GbpA fractions before purification)

Tables S1-S4 (S1, M9glyc+ minimal medium for GbpA production in BL21(DE3); S2, M9max minimal medium for GbpA production in Vmax™ X2; S3, Data collection and refinement statistics for CT produced from Vmax™ X2; S4, Quantification of GbpA deuteration)

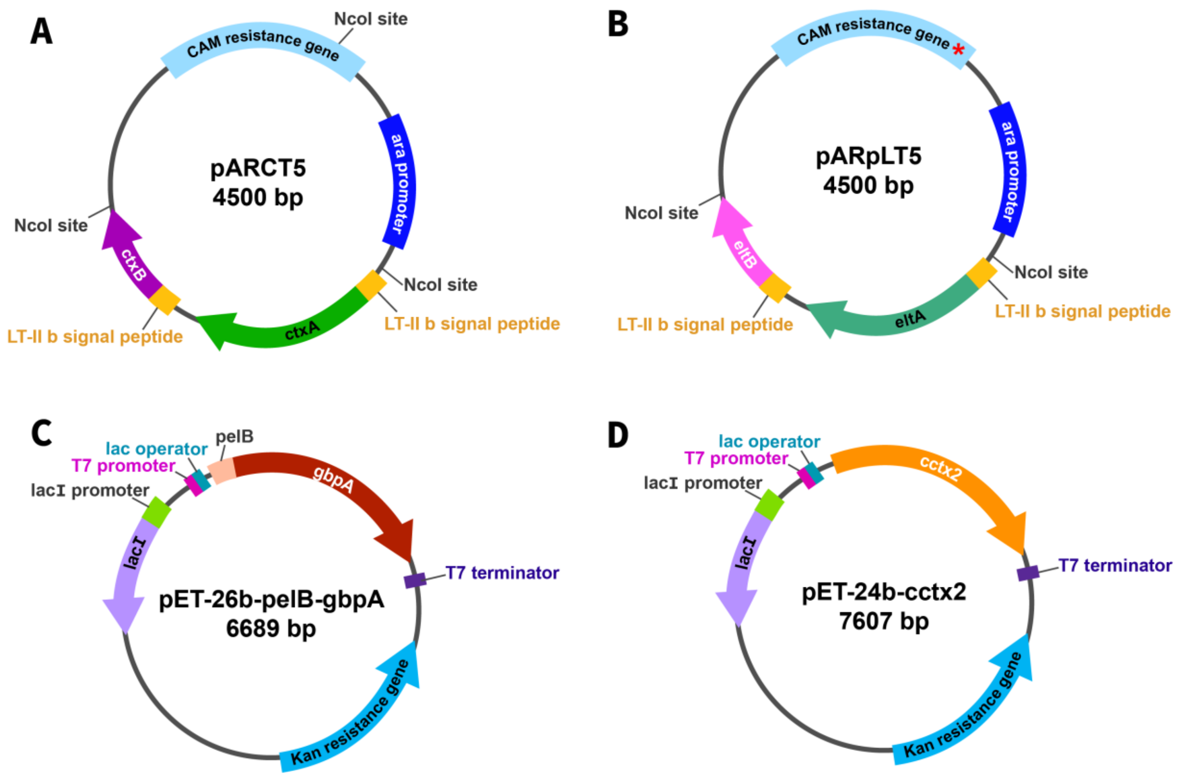

Figure S1. Plasmid maps. A *ctxAB* expression plasmid (pARCT5). **B** *eltAB* expression plasmid (pARpLT5). The red asterisk in the CAM resistance gene represents the silent mutation introduced to remove an NcoI site (leaving only two NcoI restriction sites in the vector). **C** *gbpA* expression plasmid (pET-26b-pelB-gbpA). **D** *cctx2* expression plasmid (pET-24b-cctx2)

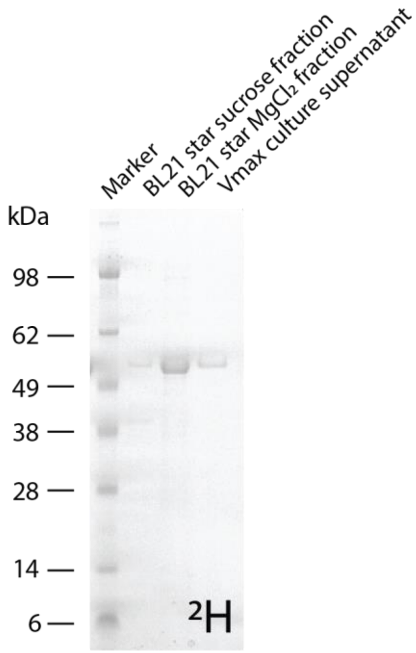

Figure S2. SDS-PAGE analysis of GbpA fractions before purification. ^{2}H is the chemical symbol for deuterium, a chemical isotope of hydrogen with one proton and one neutron (whereas common hydrogen (^{1}H) lacks neutrons).

Table S1: M9glyc+ minimal medium for GbpA production in BL21(DE3)

Minimal medium	
K_2HPO_4	109 mM
KH_2PO_4	37 mM
Na_2HPO_4	63 mM
K_2SO_4	14 mM
NH_4Cl	93 mM
$MgCl_2$[a]	10 mM
Glycerol	1.6 %
MEM vitamins[b]	1x
Trace Element solution[c]	1x

[a] Added from a stock solution just before inoculation to minimize precipitation of phosphate salts
[b] MEM 100x vitamin solution from Sigma-Aldrich
[c] The 1000x trace element solution was made by dissolving the following ingredients in 100 mL H_2O: 0.6 g $FeSO_4 \times 7H_2O$, 0.6 g $CaCl_2 \times 2H_2O$, 0.12 g $MnCl_2 \times 4H_2O$, 0.08 g $CoCl_2 \times 6H_2O$, 0.07 g $ZnSO_4 \times 7H_2O$, 0.03 g $CuCl_2 \times 2H_2O$, 2 mg H_3BO_4, 0.025 g $(NH_4)_6Mo_7O_{24} \times 4H_2O$, 0.5 g EDTA.

Table S2: M9max minimal medium for GbpA production in Vmax™ X2

Minimal medium	
Na_2HPO_4	150 mM
KH_2PO_4	75 mM
NaCl	10 mM
NH_4Cl	28 mM
$MgSO_4$[a]	5 mM
$CaCl_2$[a]	0.2 mM
Glycerol	1.6 %
MEM vitamins[b]	1x
Trace Element solution[c]	1x

[a] Added from a stock solution just before inoculation to minimize precipitation of phosphate salts
[b] MEM 100x vitamin solution from Sigma-Aldrich
[c] The 1000x trace element solution was made by dissolving the following ingredients in 100 mL H_2O: 0.6 g $FeSO_4 \times 7H_2O$, 0.6 g $CaCl_2 \times 2H_2O$, 0.12 g $MnCl_2 \times 4H_2O$, 0.08 g $CoCl_2 \times 6H_2O$, 0.07 g $ZnSO_4 \times 7H_2O$, 0.03 g $CuCl_2 \times 2H_2O$, 2 mg H_3BO_4, 0.025 g $(NH_4)_6Mo_7O_{24} \times 4H_2O$, 0.5 g EDTA.

Minimal media recipes were adapted from Cai *et al.*[23] as we described by Sørensen *et al.*[22]

Table S3. Data collection and refinement statistics for CT produced in Vmax™ X2

**Data collection**	
Beamline	ESRF-ID30B
Wavelength (Å)	0.9763
Space group	$P2_1$
Cell parameters – a, b, c (Å)	60.7, 108.1, 124.8
Protein chains in a.u.	2
Matthew's coefficient (Å^3/Da)	2.4
Resolution (Å)[a]	124.1-2.3 (2.35-2.30)
$CC_{1/2}$ (%)[a]	97.3 (17.0)
R_{merge} (%)[a]	25.6 (>100)
$R_{p.i.m}$ (%)[a]	16.9 (>100)
Mean I/$\boldsymbol{\sigma}$ (I)[a]	4.3 (0.6)
Completeness (%)[a]	98.6 (99.3)
Number of unique reflections[a]	70156 (4548)
Multiplicity[a]	3.0 (2.9)
Wilson ***B***-factor (Å^2)	38.7
**Refinement**	
R_{work}/R_{free} (%)	22.1/26.2 %
Average *B*-factor (Å^2)	44.7
Number of atoms	13002
Protein	12409
Water	354
Ligands	239
r.m.s.d from ideal geometry	
Bond lengths (Å)	0.01
Bond angles (deg.)	1.14
Ramachandran plot	
Favored (%)	96.9
Allowed (%)	3.0
Outliers (%)	0.1
PDB ID	8QRE

[a] Values in parenthesis refer to the highest-resolution shell

Table S4. Quantification of GbpA deuteration (based on non-labile hydrogens)

Molecular mass (Da)	**Theoretical**	**BL21 Star (DE3)**	**Vmax™ X2**
**Hydrogenated (^1H)**	51254	51478	51976
**Deuterated (^2H)**	53934	54045	54565
**Deuteration level**		96%	97%
